# The Functional Characterization of MaGS2 and Its Role as a Negative Regulator of *Ciboria shiraiana*

**DOI:** 10.3390/plants13121660

**Published:** 2024-06-15

**Authors:** Keermula Yidilisi, Yuqiong Wang, Zixuan Guo, Yangyang Guo, Xiaoru Kang, Shan Li, Wenhao Zhang, Nan Chao, Li Liu

**Affiliations:** 1 Jiangsu Key Laboratory of Sericultural Biology and Biotechnology, School of Biotechnology, Jiangsu University of Science and Technology, Zhenjiang 212100, China; 2 Key Laboratory of Silkworm and Mulberry Genetic Improvement, Ministry of Agriculture and Rural Affairs, Sericultural Research Institute, Chinese Academy of Agricultural Sciences, Zhenjiang 212100, China

**Keywords:** biotic stress, glutamine synthetase, mulberry, sclerotiniose

## Abstract

Glutamine synthetase (GS) is a key enzyme involved in nitrogen metabolism. GS can be divided into cytosolic and plastidic subtypes and has been reported to respond to various biotic and abiotic stresses. However, little research has been reported on the function of GS in mulberry. In this study, the full length of *MaGS2* was cloned, resulting in 1302 bp encoding 433 amino acid residues. MaGS2 carried the typical GS2 motifs and clustered with plastidic-subtype GSs in the phylogenetic analysis. MaGS2 localized in chloroplasts, demonstrating that MaGS2 is a plastidic GS. The expression profile showed that *MaGS2* is highly expressed in sclerotiniose pathogen-infected fruit and sclerotiniose-resistant fruit, demonstrating that *MaGS2* is associated with the response to sclerotiniose in mulberry. Furthermore, the overexpression of *MaGS2* in tobacco decreased the resistance against *Ciboria shiraiana*, and the knockdown of *MaGS2* in mulberry by VIGS increased the resistance against *C. shiraiana*, demonstrating the role of *MaGS2* as a negative regulator of mulberry resistance to *C. shiraiana* infection.

## 1. Introduction

Glutamine synthetase (GS; EC6.3.1.2) is a key enzyme involved in nitrogen metabolism, including the assimilation of ammonia deriving from mineral nutrition, biological nitrogen fixation, photorespiration, and amino acid breakdown, which also initially converts inorganic nitrogen from the soil into organic nitrogen through the combined action of glutamate synthase (GOGAT; EC 1.4.1.14 and EC 1.4.7.1) in higher plants [1,2]. GS has a crucial function in nitrogen metabolism as it catalyzes the assimilation of ammonium from different sources to form glutamine, so it is essential for ammonium assimilation, reassimilation, and cycling [3]. In wheat, GS2 is responsible for the reassimilation of NH_4_^+^ generated from photorespiration and the assimilation of NH_4_^+^ derived from NO_3_^−^ reduction [4]. Three GS superfamilies (GSI, GSII, and GSIII) have been widely identified in both prokaryotes and eukaryotes based on their gene/protein sequences, protein structure phylogenetic analyses, and their molecular weight. Prokaryotes are more likely to have the isoforms of GSI, while isoforms of GSII are more common in eukaryotes [5,6,7,8]. In plants, glutamine synthesis is catalyzed by enzymatic proteins belonging to the GSII superfamily, and two groups of functional GS enzymes have been found: eubacterial GSIIb (GLN2) and eukaryotic GSIIe (GLN1/GS) [6]. Phylogenetic studies have classified the different GS genes of seed plants into three clusters: two cytosolic glutamine synthetases, GS1a and GS1b, and chloroplastidic GS2. GS1a was firstly identified in pine and thought to be divergent gymnospermous GS1 [6,9]. Since then, GS1a genes have been identified in all gymnosperms, basal angiosperms, and some Magnoliidae species, excluding more recent angiosperm species [6]. GS2 has also been found in Cycadopsida gymnosperms, suggesting the origin of GS2 in a common ancestor of Cycadopsida, Ginkgoopsida, and angiosperms [2,10]. In general, two types of GS isoforms exist in higher plants: cytosolic GS1b, which is also known as GS1, and plastidic GS2. GS1 belongs to a small, multi-gene family; localizes in vascular tissues and roots; and primarily generates glutamine for intercellular nitrogen transport [11,12]. GS2, which is encoded by a single gene, is predominantly expressed in green tissues, playing an essential role in the reassimilation of the ammonium released by photorespiration [13]. 

GS plays a fundamental role in growth and development in plants [5]. The GSs in *Arabidopsis thaliana* and rice were reported to be involved in plant growth and differentiation. The role of GS in senescence had been reported in some species, including *Arabidopsis thaliana*, rice, wheat, and barley [7]. Transgenic poplars with an ectopic expression of pine cytosolic GS exhibited enhanced vegetative growth [14,15]. GS-overexpressing rice plants also showed enhanced vegetative growth with higher soluble protein concentrations in their leaves but decreased growth in terms of both grain yield production and total amino acids in their seeds [1]. In addition, GS plays important roles in the response to diverse abiotic stresses. A role for cytosolic GS was clearly established in proline production [16]. Transgenic poplar with pine cytosolic GS showed enhanced resistance to drought stress [14]. In rice, *OsGS1;2*-overexpressing plants exhibited resistance to Basta selection and higher sensitivity to salt, drought, and cold stress conditions [1]. In addition, overexpression of the *GS2* in tobacco and rice resulted in enhanced tolerance to photooxidation and to salt stress [17,18]. GS was also reported to play a role in rice susceptibility to pathogen infection. A previous study indicated that glutamine is a potential key nutrient during Nitrogen-Induced Susceptibility (NIS), and a mutant of the *OsGS1-2* glutamine synthetase gene enhanced plant resistance to *Magnaporthe oryzae* and abolished NIS [19].

Mulberry (*Morus* spp., Moraceae) is a traditional economic crop plant with ecological and nutritional value [20]. Mulberry sclerotiniose is a devastating fungal disease affecting mulberry fruit quality and yield [21,22]. *Ciboria shiraiana* is the dominant causal agent of mulberry sclerotiniose in China, and it results in hypertrophy sorosis sclerotiniose, which limits the healthy development of a diversified utilization of mulberry [23]. GS genes have been reported to be involved in plant development and resistance to diverse stresses. However, studies on GS in mulberry are few. In the present study, a plastidic subtype of glutamine synthetase *MaGS2* was identified in mulberry, and its role in the response to sclerotiniose infectious disease was explored. Finally, *MaGS2* was functionally characterized as a negative regulator for resistance to *C. shiraiana* infection in mulberry.

## 2. Results

### 2.1. MaGS2 Is a Plastidic-Subtype Glutamine Synthetase

**Figure 1:**
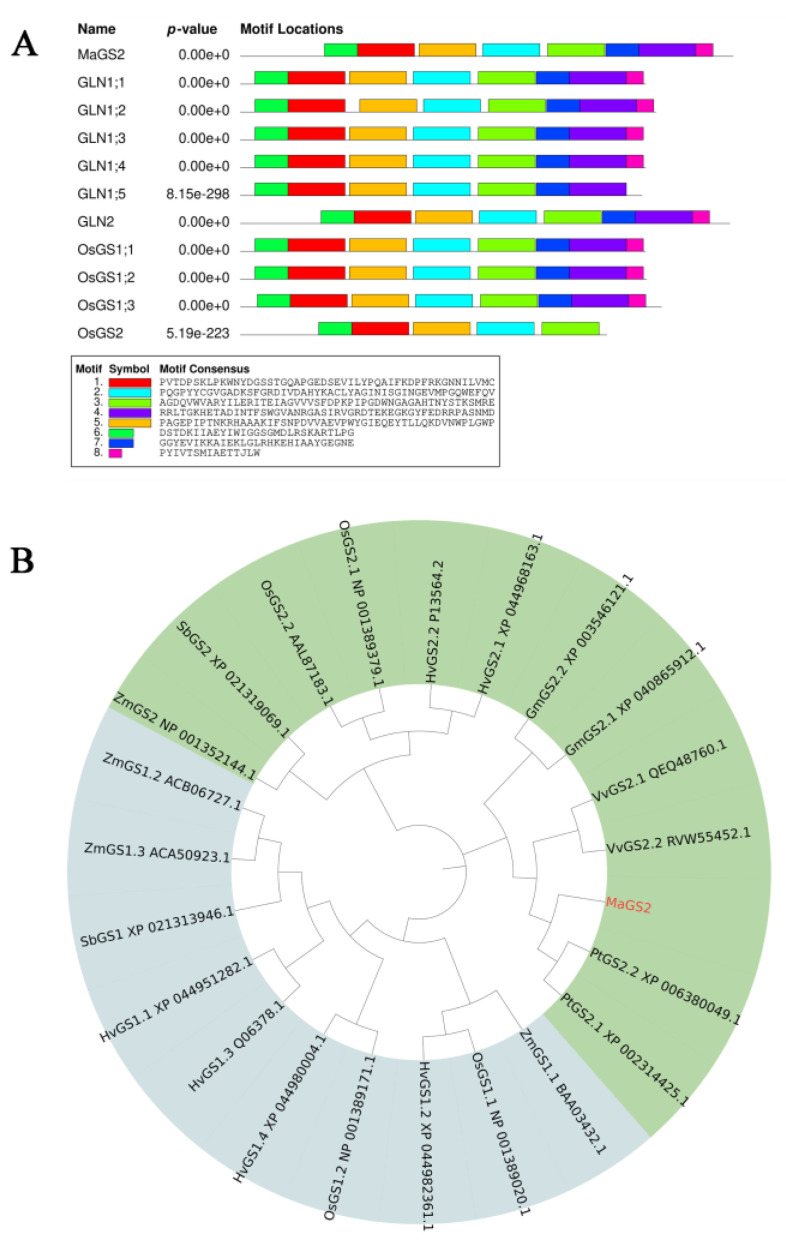
Motif analysis and phylogenetic analysis of GSs. (**A**) Motif analysis of GS in different species, including mulberry (MaGS2), *Arabidopsis thaliana* (GLN1;1, GLN1;2, GLN1;3, GLN1;4, GLN1;5, GLN2), and *Oryza Sativa* (OsGS1;1, OsGS1;2, OsGS1;3, OsGS2) (Os04g0659100). (**B**) Phylogenetic analysis of GS. The amino acid sequences of GS in mulberry, *Vitis vinifera, Oryza sativa*, *Populus trichocarpa*, *Sorghum bicolor*, *Zea mays, Hordeum vulgare*, and *Glycine max* are shown. The GS2s are highlighted with a light green background color, and the GS1s are highlighted with a light blue background.

**Figure 2:**
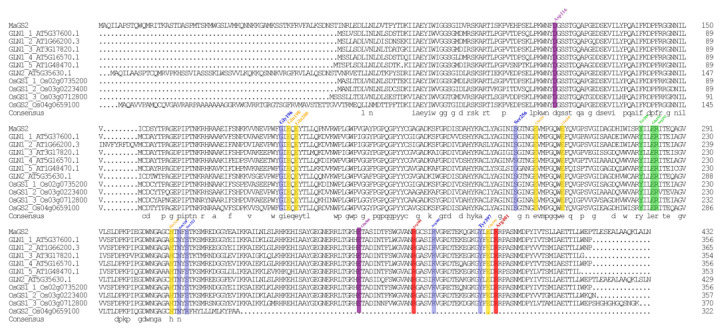
Multiple sequence alignment of MaGS2 and GSs from *Arabidopsis thaliana* and *Oryza sativa*. Gly196, Ser256, Asn320, Ser322, Arg385, and Tyr397 are associated with substrate-binding sites and are highlighted in blue. Arg380 and Arg401 are associated with the phosphate transfer reaction and are highlighted in red. Tyr288, Glu291, and Arg292 are involved in hydrophobic interactions between subunits and are highlighted in green. Glu198, Glu200, Glu261, Glu268, His318, and Glu399 are involved in cationic coordination and are highlighted in yellow. Glu366 and Asp116 are involved in the assimilation of NH_4_^+^ and are highlighted in purple.

The coding sequence of the *MaGS2* gene was cloned based on the sequence information identified in the mulberry genome database, and the sequence was deposited in GenBank with the accession number OR797682. The bioinformatics analysis showed that the full-length coding sequence of *MaGS2* was 1302 bp. *MaGS2* encodes a peptide with 433 animo acid residues; a molecular weight of 47.6 kDa; a theoretical PI of 6.8, which means that it is acidic; and an instability index of 41.76 (>40), implying the instability of the encoded protein. The multiple sequences’ alignment results showed that MaGS2 shares a 69.85% consensus with GLN1;1, GLN1;2, GLN1;3, GLN1;4, GLN1;5, and GLN2 from *Arabidopsis thaliana*, as well as OsGS1;1, OsGS1;2, OsGS1;3, and OsGS2 from rice (*Oryza Sativa*). The motif analysis using the MEME online tool showed that MaGS2 contains eight typical GS conservative motifs of chloroplast-subtype GS instead of the cytosolic subtype (Figure 1A). The phylogenetic analysis showed that GS proteins could be clustered into two subgroups, GS1 (cytosolic) and GS2 (plastidic), and MaGS2 was clustered with PtGS2.1 and PtGS2.2. PtGS2.1 and PtGS2.2 have been characterized as being of the plastidic subtype [11], indicating that MaGS2 is a plastidic-subtype GS (Figure 1B). Further, the multiple sequence alignment of amino acid sequences of GSs showed that MaGS2 contains substrate-binding sites (Gly196, Ser256, Asn320, Ser322, Arg385, and Tyr397), as well as other functional sites of typical GS (Figure 2). 

### 2.2. MaGS2 Locates in the Chloroplast

**Figure 3:**
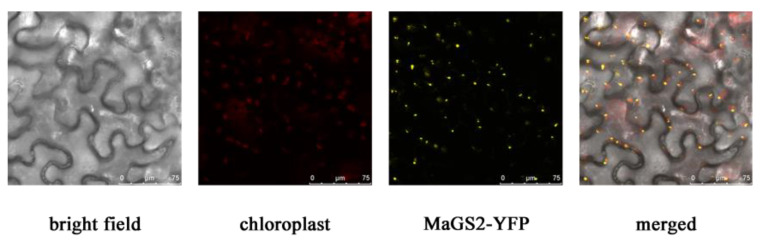
Subcellular localization of MaGS2. The red signal indicates chloroplasts, and the YFP signal overlays with the red signal, indicating that MaGS2 locates in the chloroplast.

The prediction of the subcellular localization of MaGS2 by the online tool Plant-mPLoc showed that MaGS2 localizes in the chloroplast or mitochondrion. The subcellular localization results showed that the yellow florescent protein (YFP) signal of the recombinant protein MaGS2-YFP was overlaid with the chloroplast signal (red signal) (Figure 3), which indicates that MaGS2 localizes in the chloroplast, demonstrating that MaGS2 is a chloroplastidic-subtype GS.

### 2.3. Transcriptional Characteristics of MaGS2 Are Associated with Sclerotiniose

**Figure 4:**
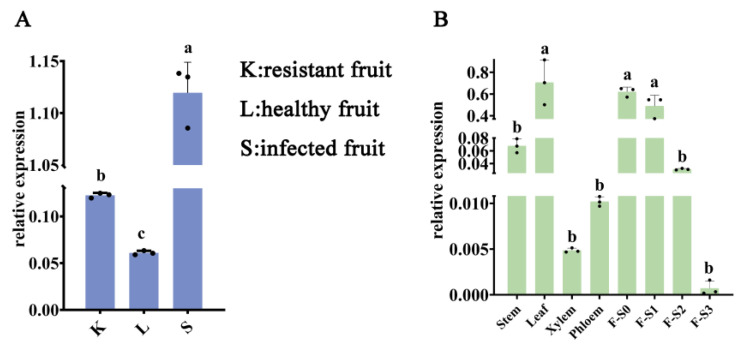
Expression profiles of *MaGS2*. (**A**) The expression levels of *MaGS2* in sclerotiniose-resistant fruit (K), healthy fruit (L), and sclerotiniose-infected fruit (S). (**B**) The expression levels of *MaGS2* in the stem, leaf, xylem, phloem, and fruits at different development stages (F-S0~F-S3). The same letter in lower case indicates no significant difference, while different letters indicate significant differences.

To understand the response of *MaGS2* to sclerotiniose, the relative expression levels of *MaGS2* in sclerotiniose-resistant fruit (K), healthy fruit (L), and sclerotiniose-infected fruit (S) were explored. The results showed that the expression level of *MaGS2* was much higher in S than in L and K, and the expression level in K was higher than that in L (Figure 4A). It is obvious that MaGS2 showed a positive response to sclerotiniose. Among the tissues, including the stem, leaf, xylem, phloem, and fruits at four different development stages (F-S0, F-S1, F-S2, F-S3), *MaGS2* was highly expressed in the leaf, F-S0, and F-S1 and barely expressed in the xylem, phloem, stem, F-S2, and F-S3 (Figure 4B). The expression level of *MaGS2* decreased with the development of the fruit. These results indicate that *MaGS2* is preferentially expressed in the leaf and unripe mulberry fruits.

### 2.4. MaGS2 Negatively Regulates Resistance against Ciboria shiraiana Infection

**Figure 5:**
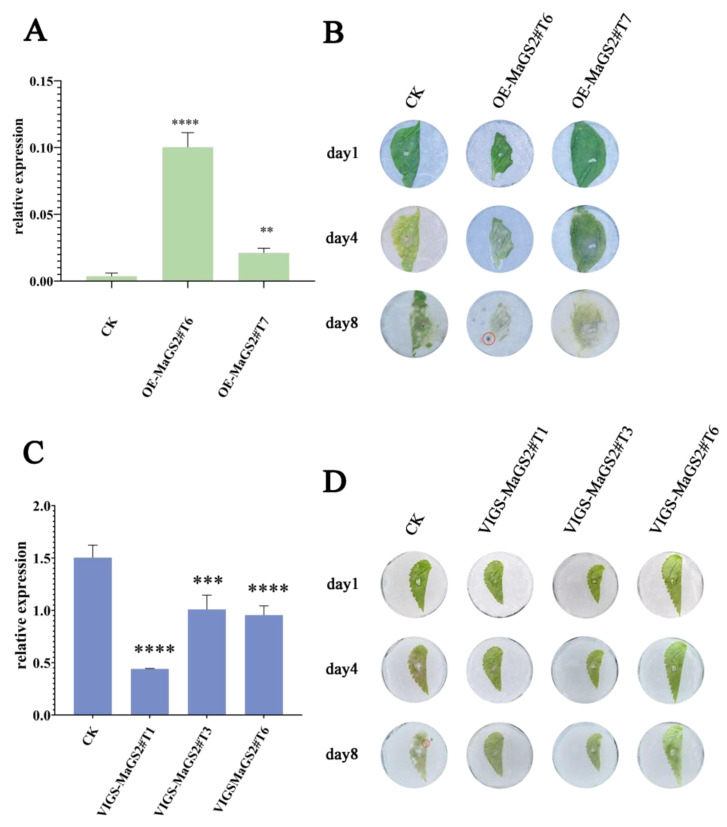
Modification of *MaGS2* expression level affects plant resistance to *C. shiraiana* (**A**) Expression level of *MaGS2* in tobacco in control (CK) and transient overexpression lines. (**B**) Infection with *C. shiraiana* after overexpression of *MaGS2* in tobacco. (**C**) Expression level of *MaGS2* in mulberry leaves treated by VIGS. (**D**) Damage of mulberry leaves with *MaGS2* knockdown after infection by *C. shiraiana*. ** represents significance levels, with ** for *p* < 0.001, *** for 0.0001 < *p* < 0.001, **** for 0.00001 < *p* < 0.0001; red circles represent sclerotium.

To understand the influence of MaGS2 on resistance against the sclerotiniose pathogen *C. shiraiana*, transient *MaGS2*-overexpressing tobaccos lines were obtained; then, the leaves of the transient *MaGS2*-overexpressing tobacco were inoculated with a PDA disc culture of *C. shiraiana*. The results showed that *MaGS2* was truly and significantly overexpressed in the treated tobacco leaves (Figure 5A). The OE-MaGS#T6 leaf was fully damaged with cell death symptoms, and the damaged area of OE-MaGS#T7 was much larger than that of the control 4 days after inoculation. In addition, sclerotium (indicated by red circles) appeared on OE-MaGS2#T6 prior to the control. The cell death symptoms of OE-MaGS2#T7 were even more severe when compared with those of the control 8 days after inoculation (Figure 5B). 

Inoculation with a PDA disc culture of *C. shiraiana* was also performed using mulberry leaves. The qRT-PCR results showed that the expression level of *MaGS2* was significantly knocked down by VIGS (virus-induced gene silencing) (Figure 5C). When inoculated with a disk culture of *C. shiraiana*, the damaged areas on MaGS2-knockdown leaves were smaller than those on control leaves 4 days after inoculation. Furthermore, sclerotium appeared on the control leaves 8 days after inoculation (Figure 5D). Altogether, these results indicate that MaGS2 is a negative regulator of resistance against *C shiraiana* infection.

## 3. Discussion

Being an indispensable member of the GS-GOGAT cycle, through which plants complete approximately 95% of their NH_4_^+^ assimilation, GS plays pivotal roles in overall N flow across the living system [7]. GS is encoded by a small family of nuclear genes. GS1 is encoded by several functional redundancy genes and usually locates in the cytosol, while GS2 is encoded by a single nuclear gene, targets the chloroplast, and is larger than GS1 in terms of its molecular weight [3,7]. In the present study, the *GS2* gene in mulberry was explored. MaGS2 contains a conserved domain similar to that of GS2 in *Arabidopsis thaliana* (Figure 2) and localizes in the chloroplast (Figure 3), indicating that MaGS2 belongs to the plastidic subtype of GS. These results are consistent with results obtained for *Arabidopsis thaliana* and wheat [3,4,7]. Studies on plastidic GS (GS2) have been reported in many seed plants and have mainly focused on its roles in plant development and in response to abiotic stresses [7,13]. In other studies, GSs were reported to mitigate the effects resulting from a range of abiotic stresses, including cold, salt, drought, light, NH_4_^+^ toxicity, H_2_O_2_ treatment, wounding, ultraviolet B radiation, heat, and oxidative stress in plant systems [7]. Few studies have focused on its role in response to biotic stresses. In this study, the *MaGS2* gene was characterized as being involved in a response to biotic stress. Additionally, the expression pattern of *MaGS2* showed tissue and development stage expression preferences, which indicates that *MaGS2* is involved in mulberry fruit development and biotic stress mediation (Figure 4AB). Furthermore, the tissues with a high expression level of *MaGS2* are also rich in chloroplasts; this could be explained by its function in the development and degeneration of chloroplasts. Mutants lacking plastidic GS are conditionally lethal, and the knockdown of GS delays DNA repair and nucleotide metabolism in both in vivo and in vitro animal systems [13,24]. The nitrogen supply strongly affects rice blast susceptibility but only slightly affects plant growth [19]. The occurrence of Nitrogen-Induced Susceptibility (NIS) implies the possible role of GS in affecting resistance to biotic stresses. In rice, a mutant of the glutamine synthetase *OsGS1-2* gene enhances plant resistance to *M. oryzae* and abolishes NIS [19]. In *Arabidopsis thaliana*, *gln2* mutants had been speculated to be lethal, but a recent study revealed that *gln2*-knockout mutants were viable and did not show any aberrant phenotypes in air or high CO_2_ [25]. However, the role of GS2 in regulating resistance to pathogen infections is still unknown. The current work identified the *MaGS2* gene in mulberry, and its expression level was significantly induced by sclerotiniose infection (Figure 4A). As reported in many plant species, *GS1* is mainly expressed in roots and vascular tissues, while plastidic *GS2* has an expression preference in leaves, which corresponds to its chloroplast localization [5,11,12,26]. MaGS2, which was determined to locate in chloroplasts, also showed its highest expression level in the leaf in mulberry. Interestingly, *MaGS2* also showed a high expression level in immature fruits at early developmental stages. It is known that the fruit ripening process occurs along with the deterioration of chloroplasts [27,28]. The decreased expression level of *MaGS2* is consistent with the decay of chloroplasts. Given that outbreaks of sclerotiniose diseases mainly occur at the early development stage of mulberry fruits, the expression pattern of *MaGS2* in developmental fruits and diseased fruits suggests its possible role in response to sclerotiniose pathogen infection. In the present study, the transient overexpression of *MaGS2* in tobacco decreased resistance against *C. shiraiana*, and the plant resistance decreased with an increased expression level of MaGS2 (Figure 5A). The knockdown of *MaGS2* in mulberry increased resistance against *C. shiraiana* (Figure 5D). Both the overexpression and knockdown of *MaGS2* suggest that *MaGS2* works as a negative regulator of mulberry resistance to *C. shiraiana*. Therefore, it is likely that *MaGS2*, like the *OsGS1-2* gene in rice, regulates mulberry resistance to fungal pathogens through NIS. Further studies should be carried out to underline the NIS mechanism regulated by *GS2* in mulberry. 

## 4. Materials and Methods

### 4.1. Plant Materials

Mature, healthy purple fruits (L) from cultivar Zhongshen No.1, sclerotiniose-infected fruits (S) from cultivar Zhongshen No.1 with the typical symptom (milky-white swollen fruit) of hypertrophy sorosis sclerotiniose, and mature healthy fruits from the sclerotiniose-resistant cultivar K were used to detect the response of *MaGS2* to sclerotiniose. Mulberry fruits in different development stages, including inflorescence (F-S0), green fruits (F-S1), reddish fruits (F-S2), and mature purple fruits (F-S3), and different tissues, including stem, leaf, phloem, and xylem, all from the cultivar Zhongshen No.1, were used to explore the expression profile of the *MaGS2* gene. All the plant materials were collected from National Mulberry Gene Bank (NMGB), Zhenjiang, China. All the plant tissues and organs were frozen immediately with liquid nitrogen and kept in a −80 °C ultra-low-temperature refrigerator until use.

Mulberry seedlings for VIGS assay and tobacco seedlings for transient overexpression were cultured in a growth chamber at 28 °C for 16 h under light and 26 °C for 8 h under dark conditions.

### 4.2. RNA Extraction and cDNA Synthesis

Plant materials were ground into powder under liquid nitrogen. The total RNA was extracted following the instructions of the easyspin Plus Plant RNA extraction toolkit (RN38) (Aidlab, Beijing, China). The RNA quality and concentration were detected by agarose gel electrophoresis and a NanoDrop One nucleic acid micro detector (Thermo Scientific, Waltham, MA, USA), respectively. cDNA was synthetized following the instructions of the Hiscript III RT supermix for qPCR toolkit (Vazyme, Nanjing, China) with 1 μg RNA.

### 4.3. Cloning and Bioinformatic Analysis of MaGS2

The CDS of *MaGS2* was amplified by PCR using specific primers named MaGS2-F and MaGS2-R (sequences of primers, from the 5′ end to the 3′ end, MaGS2-F: atggcacagattttggcac, MaGS2-R: ctagacattcaaagccagct) and then cloned into a *pMD19-T* vector (Takara, Dalian, China), followed by transformation to the *E. coli DH5α* strain (Vazyme, Nanjing) and sequencing by SUNYA biotechnology company (Hangzhou, Zhejiang). The chemical and physical characteristics of the MaGS2 protein were predicted by the online tool ProtParam (https://web.expasy.org/protparam/protparam-doc.html (accessed on 5 December 2023)). The multiple sequence alignment and conservative motif analysis were performed by DNAMAN 8.0 software (version 8.0, Lynnon Corp., Quebec, Canada) using default parameters and the MEME online tool (https://meme-suite.org/ (accessed on 7 December 2023)). Sequences including GS in *Arabidopsis thaliana* (GLN1;1 (AT5G37600.1), GLN1;2 (AT1G66200.3), GLN1;3 (AT3G17820.1), GLN1;4 (AT5G16570.1), GLN1;5 (AT1G48470.1), GLN2 (AT5G35630.1)) and in *Oryza sativa* (OsGS1;1 (Os02g0735200), OsGS1;2 (Os03g0223400), OsGS1;3 (Os03g0712800), OsGS2 (Os04g0659100)) were used. The phylogenetic analysis was performed with MEGA X software [29]; the multiple sequence alignment for the phylogenetic tree was performed by clustalW software assembled in Mega X (http://www.clustal.org/) with default parameters; and the phylogenetic tree was constructed by the Neighbor-Joining method, using the Bootstrap method to test the phylogeny, with 1000 replicates and the p-distance model for substitution. The phylogenetic tree was colored using the online tool ITOL (https://itol.embl.de/ (accessed on 10 December 2023)).

### 4.4. Transcriptional Characteristics of MaGS2

The transcriptional characteristics of the *MaGS2* gene were obtained by qRT-PCR with the ABI StepOnePlus™ Real-Time PCR System (Foster City, CA, USA). The expression levels of the *MaGS2* gene in the fruit of sclerotiniose-resistant cultivar K (K), healthy fruit of cultivar Zhongshen No.1 (L), and sclerotiniose-infected fruit of cultivar Zhongshen No.1 (S) were detected to understand the response of *MaGS2* to sclerotiniose. The expression levels of *MaGS2* in tissues, including the stem, leaf, xylem, phloem, and fruits in four development stages (F-S0, F-S1, F-S2, F-S3), were detected to understand the expression preference of *MaGS2*. The specific primers for qRT-PCR were designed with Primer Premier 6.0 software and the Primer-BLAST online tool (https://www.ncbi.nlm.nih.gov/tools/primer-blast/index.cgi (accessed on 12 December 2023)) and were named MaGS2-QF and MaGS2-QR (sequences of primers, MaGS2-QF: CCTCCGCCACAAAGAGCATA, MaGS2-QR: TGAGGTCACGATGTAGGGGT). A qRT-PCR mixture containing chamQ SYBR qPCR Master Mix (High ROX Premixed) (Vazyme, Nanjing), 0.5 μM of each specific primer, and appropriate cDNA diluted with DEPC-treated ddH_2_O was prepared with a total volume of 10 μL. A two-step thermal cycling protocol (95 °C for 30 s, followed by 40 cycles of 95 °C for 5 s and 60 °C for 30 s) was adopted to perform the qRT-PCR. *MaActin* (primers: MaActin-QF and MaActin-QR) was used as an internal reference [30], and the relative expression levels of the *MaGS2* gene were computed with the 2^−ΔCt^ method. 

### 4.5. Subcellular Localization of MaGS2

Before the experiments were carried out, the subcellular localization of MaGS2 was predicted by the online tool Plant-mPLoc (http://www.csbio.sjtu.edu.cn/bioinf/plant-multi/ (accessed on 14 December 2023)) as the requirement of the developer [31]. The full-length coding sequence of *MaGS2* without the terminal codon was cloned into a pBI121 vector containing the YFP gene between the recognition sites of *Bam*H I and *Xba* I by seamless cloning. Then, the recombinant plasmids were transformed into the *E. coli DH5α* strain, followed by sequencing by SUNYA biotechnology company. The correct recombinant plasmids were transformed into the *Agrobacterium tumefaciens GV3101* strain. The transformant was cultured at 28 °C and 200 rpm with liquid LB medium containing 50 μg/mL kanamycin and rifampicin until the OD_600_ reached approximately 0.6. The culture was collected by centrifugation at 6000 rpm for 6 min. Then, the sediment pellets were resuspended with infiltration medium (10 mm MES monohydrate pH 5.6, 10 mm MgCl_2_, 200 μM acetosyringone, 400 mg/L L-Cysteine, 5 mL/L Tween-20, 50–500 μL/L Silwet L-77, 5% sucrose) and kept still for 3 h at 28 °C, after which they were infiltrated into *Nicotina benthamiana* leaves. The leaves were harvested after 48 h, and the fluorescence signal was observed by a Leica TCS SP8 confocal microscope. 

### 4.6. Overexpression and VIGS of MaGS2 Followed by C. shiraiana Infection

*MaGS2* was cloned into a pNC-Cam1304-MCS35S plasmid vector by seamless cloning and transformed into *A. tumefaciens GV3101* competent cells. The transformant was cultured at 28 °C and 200 rpm with liquid LB medium containing 50 μg/mL kanamycin and rifampicin until the OD_600_ reached approximately 0.6. The culture was collected and then resuspended with infiltration medium as described above. After stewing at 28 °C for 4 h in darkness, the infiltration medium containing the transformants was subsequently infiltrated into the leaf of *N. benthamiana*, and *A. tumefaciens* containing empty pNC-Cam1304-MCS35S vector was used as a control. The tobacco was kept in darkness for 48 h, then cultivated in a growth chamber under general conditions. The leaves of the treated tobacco were harvested after 3 days, and the leaves were cut into two halves along the vein for qRT-PCR and *C. shiraiana* infection experiments. 

For VIGS assay, the best interfering region was predicted by the SGN VIGS Tool online tool (https://vigs.solgenomics.net (accessed on 13 December 2023)), and then the best interfering region was cloned into the pNC-TRV2-GFP vector by seamless cloning, followed by transformation into *A. tumefaciens GV3101* strain competent cells. The transformant was cultivated, collected, and resuspended as described above. A 1:1 mixture of *A. tumefaciens* containing pNC-TRV2-GFP-MaGS2 and pTRV1 was infiltrated into the leaves of 4-week-old mulberry seedlings of cultivar *Fengchi* under vacuum (−0.4 to −0.6 kg/cm^2^) conditions for 4 min. A 1:1 mixture of *A. tumefaciens* containing pNC-TRV2-GFP empty vector and pTRV1 was used as a control. The treated mulberry seedlings were kept in darkness for 24 h, then cultivated under general conditions in a growth chamber. The leaves of the treated mulberry seedlings were harvested after 23 days, and the leaves were cut into two halves along the vein for qRT-PCR and *C. shiraiana* infection experiments. 

*C. shiraiana* was cultivated on potato dextrose agar medium (Coolaber, Beijing) at 26 °C for 4–5 days, then cut into small discs and put on lightly wounded half-leaves that had been sterilized with 75% ethanol for 2 min and washed with ddH_2_O. The damaged areas with cell death symptoms caused by infection and the appearance of sclerotia were observed and recorded. 

## 5. Conclusions

In this study, *MaGS2* was confirmed to be a plastidic-subtype GS by motif analysis and phylogenetic analysis, further validated by the fact that *MaGS2* localized in the chloroplast. *MaGS2* showed a variable expression profile among healthy fruit, sclerotiniose pathogen-infected fruit, and sclerotiniose-resistant fruit, revealing its close association with sclerotiniose. By the inoculation of *C. shiraiana* on the leaves of *MaGS2*-overexpressed tobacco and *MaGS2*-knocked down mulberry, MaGS2 was characterized as a negative regulator of plant resistance to *C. shiraiana* infection. All our results provide an important cue for understanding the roles of plastidic GS in the defense against sclerotiniose in mulberry. 

## Data Availability

The original contributions presented in this study are included in the article; further inquiries can be directed to the corresponding author.
